# Serotonin Depletion-Induced Maladaptive Aggression Requires the Presence of Androgens

**DOI:** 10.1371/journal.pone.0126462

**Published:** 2015-05-15

**Authors:** Erik Studer, Jakob Näslund, Erik Andersson, Staffan Nilsson, Lars Westberg, Elias Eriksson

**Affiliations:** 1 Department of Pharmacology, Institute of Neuroscience and Physiology, Sahlgrenska Academy, University of Gothenburg, POB 431, SE 405 30 Gothenburg, Sweden; 2 Mathematical Sciences, Chalmers University of Technology, SE 412 96 Gothenburg, Sweden; Brock University, CANADA

## Abstract

The sex hormone testosterone and the neurotransmitter serotonin exert opposite effects on several aspects of behavior including territorial aggression. It is however not settled if testosterone exerts its pro-aggressive effects by reducing serotonin transmission and/or if the anti-aggressive effect of serotonin requires the presence of the androgen. Using the resident intruder test, we now show that administration of the serotonin synthesis inhibitor *para*-chlorophenylalanine (300 mg/kg x 3 days) increases the total time of attack as well as the percentage amount of social behavior spent on attack but not that spent on threat – i.e. that it induces a pattern of unrestricted, maladaptive aggression – in gonadectomized C57Bl/6 male mice receiving testosterone replacement; in contrast, it failed to reinstate aggression in those not given testosterone. Whereas these results suggest the pro-aggressive effect of testosterone to be independent of serotonin, and not caused by an inhibition of serotonergic activity, the pCPA-induced induction of maladaptive aggression appears to require the presence of the hormone. In line with these findings, pCPA enhanced the total time of attack as well the relative time spent on attacks but not threats also in wild-type gonadally intact male C57Bl/6 mice, but failed to reinstate aggression in mice rendered hypo-aggressive by early knock-out of androgen receptors in the brain (AR^NesDel^ mice). We conclude that androgenic deficiency does not dampen aggression by unleashing an anti-aggressive serotonergic influence; instead serotonin seems to modulate aggressive behavior by exerting a parallel-coupled inhibitory role on androgen-driven aggression, which is irrelevant in the absence of the hormone, and the arresting of which leads to enhanced maladaptive aggression.

## Introduction

The male sexual hormone testosterone and the brain neurotransmitter serotonin exert opposite effects on many aspects of behavior. Thus, whereas testosterone has been shown to promote territorial behavior (such as urine spraying) [1], impulsivity (as assessed using various conflict paradigms) [2,3] and sexual behavior [4] in rodents, serotonin appears to exert the opposite effects on all these aspects of behavior [5–7]. Likewise, whereas lack of testosterone often impairs aggressive behavior, serotonin is reported to reduce aggression, and particularly to counteract so-called maladaptive aggression [8].

This striking functional antagonism between testosterone and serotonin with respect to behavior, in conjunction with the observation that testosterone exerts a profound influence on brain serotonergic activity [9–14], makes it tempting to suggest that the steroid might exert its behavioral effects by reducing the activity of serotonergic pathways. According to this view, the reduction in aggression and sexual activity observed in androgen-deficient animals would be the result of an anti-aggressive influence of serotonin being unleashed by the removal of the androgen. Alternatively, however, androgen-dependent behavior, including aggression, may be under the influence of a parallel, independent negative modulation by serotonin; to dampen or modulate sex steroid-driven behavior may hence constitute an important physiological role of this transmitter [15,16]. Finally, a third possibility would be that serotonin influences aspects of behavior that also happen to be under the influence of sex steroids, but would exert this influence also in the absence of the hormone.

Although many previous reports have discussed the influence of testosterone and serotonin, respectively, on the regulation of aggression, as well as the possible interplay between the two [13,17–23], there are, to our knowledge, no previous reports examining to what extent aggression induced by testosterone at physiological doses in gonadectomized (GDX) animals is serotonin-independent, i.e. if testosterone promotes aggression also in the absence of serotonin, which would not be the case if testosterone stimulates aggression mainly by dampening a tonic inhibitory serotonergic influence. Reciprocally, it is also not known if presence of testosterone is an indispensable prerequisite for the well-established aggression-provoking effect of arresting serotonergic transmission.

The resident intruder (RI) paradigm is an animal model reflecting offensive, territorial aggression in which testosterone and serotonin have previously been shown to exert pro-and anti-aggressive effects, respectively [11,22]. Aggressive behavior exerted by the resident versus the intruder in this test might be viewed as a form of functional social communication and should, as such, follow certain patterns. To fulfill its purpose it should not comprise an excessive number of actuals attacks when threats of attacks would be sufficient for the aim to be met, i.e. for eliciting submissive postures from the intruder signaling maintained territorial control and dominance by the resident. Moreover, the behavior should not continue once this goal has been reached.

In contrast, aggression characterized by a relative paucity of threat signals versus actual attacks and short latency to attack have recently been described as *maladaptive* [8]. While recent studies suggest that serotonin dampens such maladaptive (rather than functional) aggression, there are, to our knowledge, no studies exploring if the aggression induced by serotonin depletion in the resident intruder paradigm is largely of a maladaptive, dysfunctional kind, i.e. if arresting serotonergic activity elicits more attacks than threats.

The first part of this study had two purposes. Firstly we wanted to address three different possibilities with respect to the interplay between serotonin and testosterone in the regulation of aggression: *i)* that testosterone promotes aggression by inhibiting serotonergic transmission, *ii)* that serotonin instead exerts a parallel inhibitory influence on testosterone-dependent aggression (of relevance only in the presence of the androgen) and *iii)* that serotonin dampens aggression regardless of whether testosterone in present or not. Secondly we wanted to address the hypothesis that aggression induced by removal of serotonin primarily is of a maladaptive kind, i.e. that serotonin depletion promotes attacks rather than threats.

To this end, mice were rendered non-aggressive by means of orchidectomy and then exposed to implantation of slow-release testosterone pellets or sham pellets, followed by the administration of either vehicle or the irreversible inhibitor of tryptophan hydroxylase, *para*-Chlorophenylalanine (pCPA), at a regimen previously shown to effectively deplete brain serotonin [24] as well as to increase aggression [25]. If the pro-aggressive effect of testosterone were mediated by a direct or indirect reduction of an anti-aggressive serotonergic input, one would expect serotonin depletion to reinstate aggression in gonadectomized (GDX) mice; moreover, administration of testosterone would fail to cause any further increase in aggression in pCPA-treated animals (when compared to pCPA-treated GDX mice). On the other hand, if serotonin reduces testosterone-dependent aggression by a parallel inhibitory path, serotonin depletion would not be sufficient to reinstate aggression after gonadectomy, and pCPA would enhance rather than mask the pro-aggressive effect of the hormone. Finally, if serotonin dampens testosterone-independent aggression, pCPA would be expected to enhance aggression in GDX mice regardless of whether they are the subject of testosterone replacement or not. Also, by examining if pCPA enhances actual attacks rather than threats, we were able to address the theory that a primary role of serotonin in this context is to dampen maladaptive rather than functional aggression.

Although the pro-aggressive effects of testosterone partly seems to be mediated by its metabolite estradiol acting via estrogen receptors (ER) [26], also AR activation is required for normal aggressive behavior [27] as illustrated, e.g., by previous studies on male mice lacking functional ARs in the central nervous system (AR^NesDel^) [28,29]. In a second experiment, we wanted to shed further light on the possible interactions between serotonin and androgens for the regulation of aggression by exploring if the reduction in aggression following this form of reduced androgenicity is serotonin-dependent; to this end, it was examined if pCPA can reinstate aggressive behavior in AR^NesDel^ mice or if serotonin depletion enhances aggression in wild-type controls only. Moreover, it was again assessed if aggression provoked by pCPA is of a maladaptive kind.

## Materials and Methods

### 2.1 Animals

#### Experiment I

Male C57Bl/6N mice (Charles River, Denmark), aged 10 weeks at arrival, were anaesthetized with a 3:12 vol/vol mixture of ketamine (Ketalar 10mg/ml, Pfizer) and xylazine (Rompun Vet 20 mg/ml, Bayer Animal Health), gonadectomized via a midline incision, and allowed to recover in groups of five. After a period of two weeks, aimed at allowing hormonal levels to decline and the effect of orchidectomy on aggressive behavior to be fully developed [30], the mice were anaesthetized as described above, and implanted with slow-release pellets containing 15 mg of testosterone (T, n = 18) or vehicle (GDX, n = 15) and designed for 60 days of even release (250 μg/day) (Innovative Research of America, USA). After this operation, the animals were allowed to recover for two weeks before they were moved to individual cages. After nine days of isolation, the first RI test was undertaken.

#### Experiment II

Generation of AR^NesDel^ has been described in detail elsewhere [28]. In brief, male C57Bl/6 mice expressing Cre driven by the neuronal Nestin promoter were obtained from Jackson Laboratory (Bar Harbor, Maine, US) and mated with female mice carrying LoxP-sites [31] flanking the second exon of the androgen receptor gene which had been backcrossed into the C57Bl/6 background for at least 6 generations prior to the arrival at our laboratory [32]. Genotypes were confirmed with PCR using the following primers: AR: 5´-AGC CTG TAT ACT CAG TTG GGG- 3´ and 5´-AAT GCA TCA CAT TAA GTT GAT ACC- 3´, Cre: 5´-GTTCGCAAGAACCTGATGGACA-3´and 5´-CTAGAGCCTGTTTTGCACGTTC-3´, Zfy: 5´- AAG ATA AGC TTA CAT AAT CAC ATG GA—3´ and 5´- CCT ATG AAA TCC TTT GCT GCA CAT GT—3´. In total eight AR^NesDel^ mice were available for the experiment. As controls we used 11 littermate wild-type mice and eight AR-flox mice bearing only the LoxP-site but not expressing cre (AR^loxP^); both these groups should have intact AR activity, and they have previously been shown to display similar levels of aggression [29]. As expected, there were no differences between wild-type and AR^loxP^ with respect to any of the behavioral parameters so these groups were collapsed and used as one AR-intact control group in the statistical analyses. The animals were six to eight months of age when tested. They were moved to individual housing 24 days prior to the first tests of aggressive behavior.

#### Housing of animals

In both experiments, animals were kept under controlled conditions at the Laboratory for experimental biomedicine at the University of Gothenburg. They were housed in standard vivarium cages (375 x 215 x 150 mm) with free access to food and water on a 12/12 light/dark cycle.

### 2.2 pCPA treatment

In experiment I, gonadectomized mice given testosterone replacement as well as those without such replacement were administered either saline or pCPA methyl ester hydrochloride (300 mg/kg in saline solution buffered to pH 6) intraperitoneally once daily for three consecutive days. In experiment II, the same treatment was given to AR^NesDel^ mice and wild type controls, respectively.

### 2.3 Resident intruder test

Animals were subjected to a standard RI test as previously described [29]. Briefly, the mice were tested during the dark phase in their home cage against an intruder mouse for 15 minutes. As intruders served male mice of the 129/SvEv strain (Taconic Farms, Denmark) that were kept group-housed throughout the experiment. The intruders were weighed before each behavioral test to ensure that they were at least 0.5 g lighter than the resident, with each intruder used once daily. The test was performed twice, first to obtain a baseline assessment before drug administration, and then again 24 h after the last injection of pCPA or saline, respectively. The tests were videotaped with an overhead video camera under IR illumination for subsequent behavioral analysis.

### 2.4 Ethics

All procedures were subjected to approval by the Ethical Committee on Animal Experiments, Gothenburg, Sweden (permit numbers: 34–11, 67–12) and performed accordingly. All surgery was performed under general anesthesia and animals were allowed to recover in temperature-controlled cages, with additional monitoring for 3 days to ensure complete recovery.

### 2.5 Data analysis

Scoring was performed at low video speed by a trained observer blind to the status of the mice. Duration of all social exploration (including sniffing on the body or head of the intruder, ano-genital sniffing, social grooming and following), threat behaviors (including tail rattling, all offensive postures and aggressive grooming) and attack behaviors (including bite, chase and wrestling) were recorded. Mice not displaying at least one episode of attack or threat were characterized as non-aggressive. In addition, mice of different groups were compared with respect to total duration of attacks as well as latency to first attack; if no attack could be detected during the entire duration of the test, attack latency was set to 900 seconds. Finally, the behavioral pattern of the animals was revealed by calculating the percentage of each category of behavior (i.e. social exploration, threat and attack, respectively) in relation to the total time of social interaction [33].

### 2.6 Statistics

Fisher’s exact test was used to compare testosterone treated GDX mice versus vehicle treated GDX mice (experiment I) and wild-type mice versus AR^NesDel^ mice (experiment II) with respect to the number of mice displaying aggressive behavior (as defined in the section 2.5). In addition, comparison between the groups with respect to duration of attacks and latency to attack at the first test of each experiment was undertaken using unpaired t-test, or Welch's test for unequal variance when such variances between the groups were revealed by Levene’s test.

Since pCPA failed to evoke aggression both in GDX mice with no testosterone replacement and in AR^NesDel^ mice, a further analysis of the possible effect of serotonin depletion on aggression in the second test was performed only for testosterone-treated GDX mice of experiment I and wild-type mice of experiment II.

For both experiments, possible differences with respect to total time of attacks and latency to attack between animals treated with pCPA and saline, respectively, were analyzed using a general linear model for between-subject effects with the behavior in the first test as a covariate predictor. In addition, to explore to what extent pCPA induced maladaptive aggression as indicated by an increase in attacks rather than threats, the relative time spent on various forms of social behavior (social exploration, threat and attack, respectively) was calculated and the behavioral pattern displayed at baseline compared to that displayed after treatment with pCPA or saline. In order to separate effects of repeated testing and treatment, respectively, a linear mixed model, with test and treatment as independent factors, was used for this purpose.

Throughout the paper data are presented as group means±S.E.M.

## Results

### 3.1 Effect of treatments reducing androgen levels or androgen receptor expression on aggressive behavior

As expected, at test 1 GDX mice not receiving testosterone replacement displayed aggression to a very limited degree, only 1 out of 15 being defined as aggressive. In contrast, in the group receiving testosterone replacement, 16 out of 18 displayed aggressive behavior (Table 1A). This difference in number of mice being aggressive was reflected also by a difference with respect to duration of attack (GDX: 5.3±5.3 s, n = 15, GDX+T: 31.0±7.4 s, n = 18; t_29.6_ = -2.8; p<0.01), number of attacks (GDX: 1.5±1.5, n = 15; GDX+T: 12.8±3.1, n = 18; t_23.9_ = -3.3; p<0.01) and latency to attack (GDX: 876±23.8 s, n = 15; GDX+T: 369±70.8 s, n = 18; t_20.7_ = -6.8; p<0.001).

**Table 1:** Aggressive behavior in Experiment I.

**A)**	**Testosterone** (n = 18)		**Vehicle** (n = 15)	
No. aggressive mice (%)	16/18 (89)		1/15 (7)^###^	
Time attacking (s)	31.0 ±7.4		5.3 ±5.3^##^	
**B)**	**Saline** (n = 9)	**pCPA** (n = 9)	**Saline** (n = 8)	**pCPA** (n = 7)
No. aggressive mice (%)	9/9 (100)	9/9 (100)	1/8 (12.5)***	0/7 (0)***

**A)** Number of testosterone or vehicle treated GDX mice displaying aggression in the first resident intruder test and the total duration of attack for each group. **B)** Number of mice displaying aggression in the second resident intruder test conducted 24 hours after the final injection with saline or pCPA.

^#^ indicates level of significance versus testosterone treated mice;

^##^ p < 0.01,

^###^ p < 0.001.

* indicates level of significance versus the corresponding group of testosterone treated mice;

*** p < 0.001.

While the number of mice displaying aggression at test 1 in experiment II did not differ between groups (Table 2A), AR^NesDel^ differed from controls both with respect to time attacking (AR^NesDel^: 7.2 ±4.0 s, n = 8; controls: 45.2±12.1 s; n = 20; t_22.6_ = −3.0; p<0.01) and with respect to attack latency (AR^NesDel^: 672±109 s; n = 8; t_26_ = 2.2; controls: 372±75.7 s; n = 20; p<0.05); with respect to total number of attacks, this difference however did not reach the level of statistical significance (controls: 8.4±2.3, n = 20; AR^NesDel^: 2.9±1.5, n = 8; t_26_ = -1.5; p>0.05).

**Table 2:** Aggressive behavior in Experiment II.

**A)**	**Control** (n = 20)		**AR** ^**NesDel**^ (n = 8)	
No. aggressive mice (%)	15/20 (75)		4/8 (50)	
Time attacking (s)	45.2 ±12.1		7.2 ±4.0^#^	
**B)**	**Saline** (n = 10)	**pCPA** (n = 9)^a^	**Saline** (n = 4)	**pCPA** (n = 4)
No. aggressive mice (%)	8/10 (80)	9/9 (100)	0/4 (0)*	0/4 (0)*

**A)** Number of untreated control mice (WT and AR^loxP^) and AR^NesDel^ mice displaying aggression in the first resident intruder test and total duration of attack for each group. **B)** Number of mice displaying aggression in the second resident intruder test conducted 24 hours after the final injection with saline or pCPA.

^#^ indicates level of significance versus controls; ^#^ p < 0.05

* indicates level of significance versus the corresponding group of controls; * p < 0.05

^a^ One mouse in the pCPA treated group died prior to the second test of aggression.

### 3.2 Effect of pCPA on aggressive behavior in mice with reduced androgen levels or reduced androgen receptor expression

None of the 7 GDX animals without testosterone replacement displayed aggression after treatment with pCPA (Table 1B). Likewise, pCPA failed to provoke aggression in AR^NesDel^ mice (Table 2B).

### 3.3 Effects of pCPA on aggressive behavior in testosterone-treated GDX mice in experiment I and in wild-type mice in experiment II

**Fig 1:**
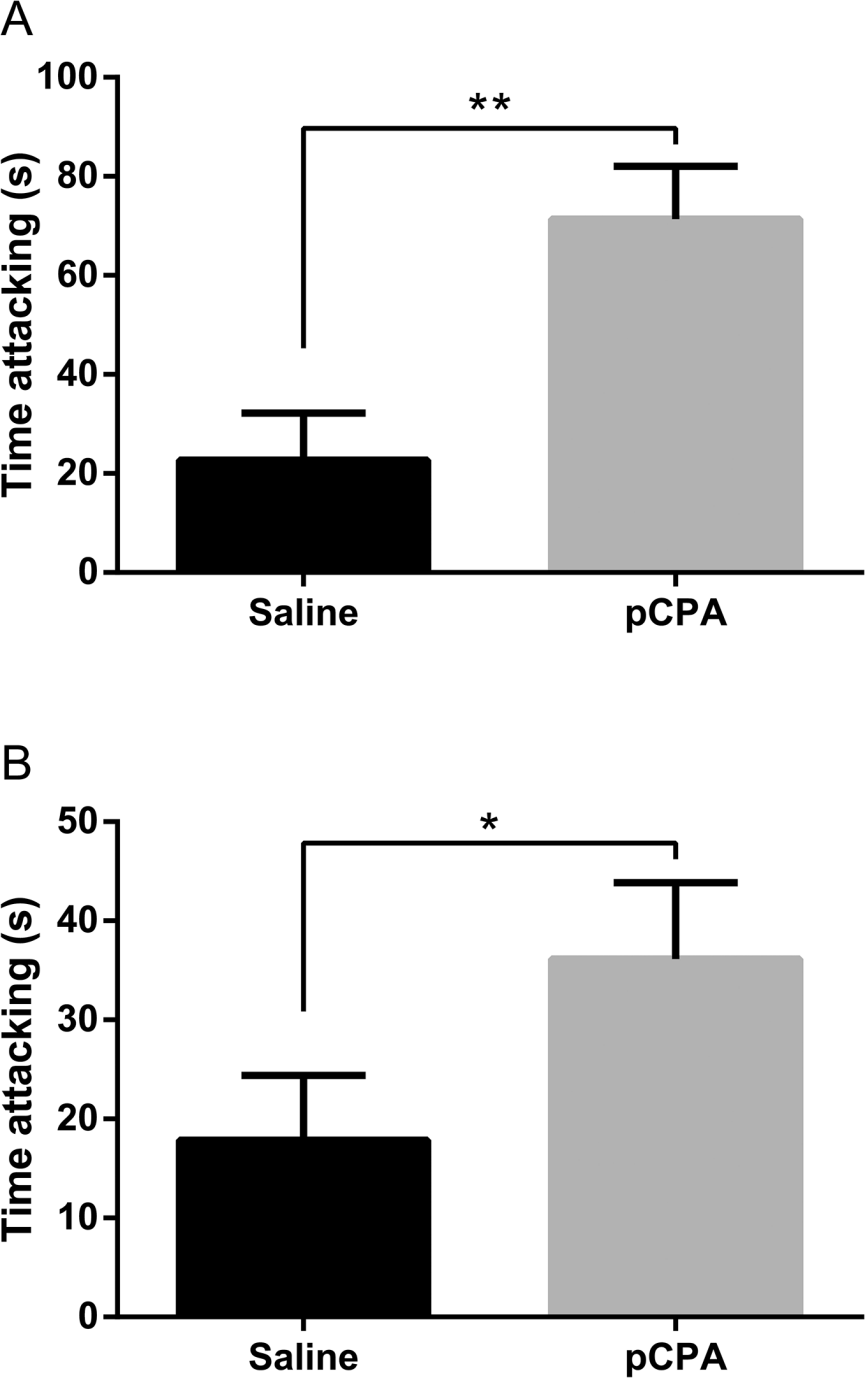
Mean duration of attacks (±SEM) in mice given saline or pCPA (300 mg/kg/day for three days). The test lasted for 15 min. A. Testosterone-treated gonadectomized mice (saline: n = 9, pCPA: n = 9) (experiment I). B. Wild-type mice (saline: n = 10, pCPA: n = 9) (experiment II). * p < 0.05, ** p < 0.01 compared to mice treated with saline (general linear model).

While pCPA failed to increase aggression in GDX mice not replaced with testosterone (experiment I) as well as in AR^NesDel^ mice (experiment II), a general linear model revealed pCPA treatment to increase total time of attack in testosterone-replaced GDX mice in experiment I (F_1,15_ = 11.6; p<0.01; Fig 1A) and in wild-type mice in experiment II (F_1,16_ = 5.1; p<0.05; Fig 1B). However, while pCPA also shortened latency in experiment II (saline: 442.2±124.7 s; n = 10; pCPA: 78.7±17.9 s; n = 9; F_1,16_ = 11.8, p<0.01), the corresponding difference did not reach significance in experiment I (saline: 215.1 ±103.8 s; n = 9; pCPA: 64.4 ±21.8 s; n = 9; F_1,15_ = 1.9; p = 0.19).

**Fig 2:**
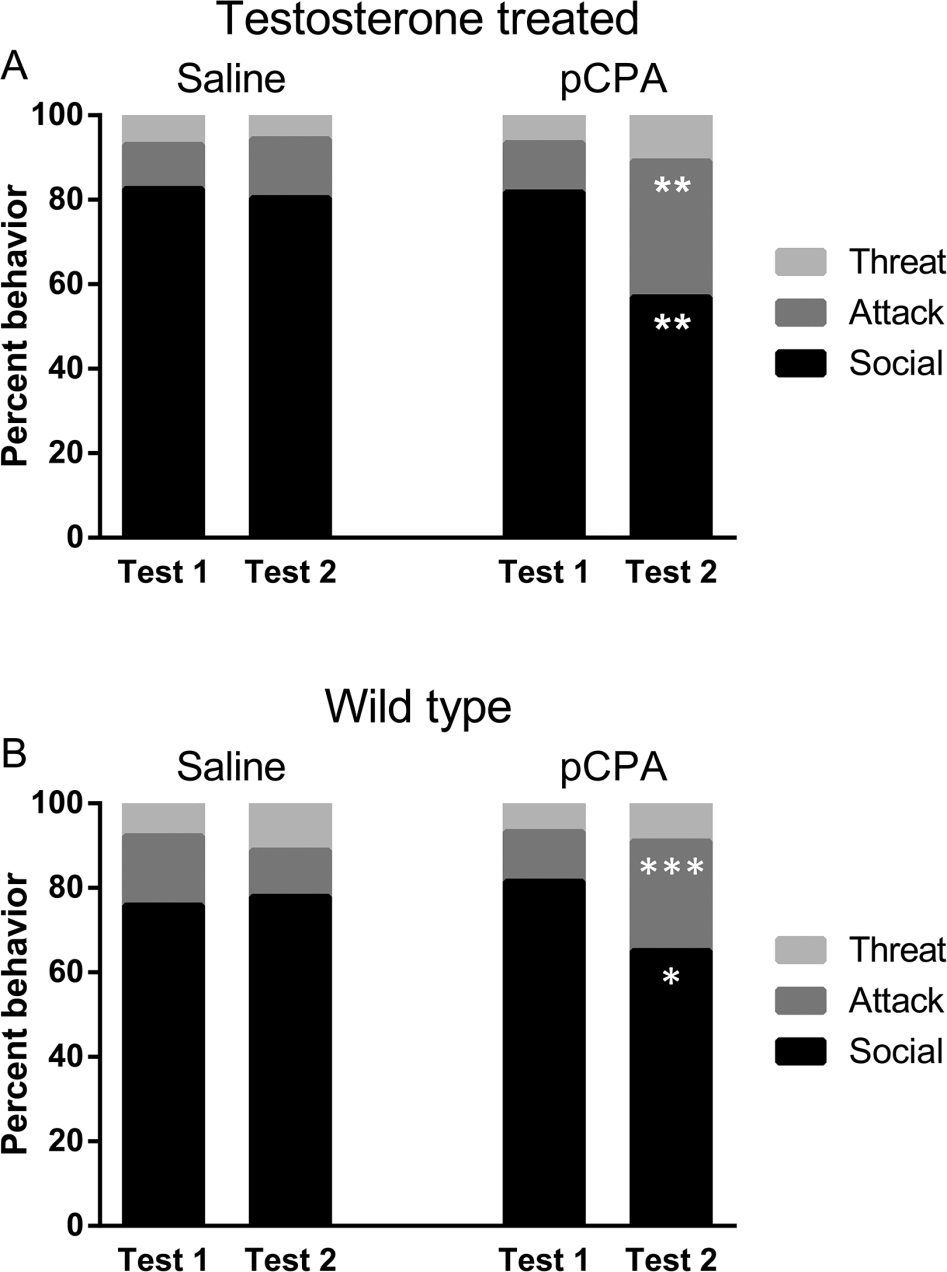
Comparison between the resident intruder baseline test (Test 1) and post-treatment test (Test 2) in animals receiving saline or pCPA (300 mg/kg/day for three days) with respect to mean percentage of the total time of social interaction spent on threat, attack and social behavior, respectively. The tests lasted for 15 min. A. Testosterone-treated gonadectomized mice (saline: n = 9, pCPA: n = 9) (experiment I). B. Wild-type mice (saline: n = 10, pCPA: n = 9) (experiment II). * p < 0.05, ** p < 0.01, *** p < 0.001 against mice treated with (linear mixed model).

A mixed model analysis regarding the relative time spent on various forms of social behavior before or after treatment with pCPA or saline revealed pCPA to induce a pattern of maladaptive aggression in both testosterone-treated GDX mice (experiment I) and in wild type mice (experiment II). In experiment I, the relative amounts of attacks but not threats were increased by serotonin depletion (% threat, t(29.8) = 1.8, p>0.05; % attack, t(31.9) = 3.0, p<0.01; % social, t(31.1) = −2.7, p<0.01; Fig 2A), and a similar effect was observed also in wild type mice in experiment II (% threat, t(32.8) = −0.4, p>0.05; % attack, t(22.0) = 3.9, p<0.001; % social, t(21.8) = −2.4, p<0.05; Fig 2B). There was no effect of repeated testing in any of the experiments.

## Discussion

In line with previous studies, orchidectomized mice displayed very low levels of aggression [30] as compared to GDX animals exposed to testosterone replacement at a dose chosen to produce serum testosterone levels in the upper physiological range [34]. This difference being observed also in mice lacking serotonin by means of pCPA administration refutes the possibility that testosterone promotes aggression by dampening an anti-aggressive serotonergic input. We hence conclude that serotonergic neurons, rather than being the mediator of the influence of testosterone on aggression, exert a dampening influence on aggression that is parallel to the aggression-promoting influence of the androgen. Further, the absence of pro-aggressive effect of pCPA in GDX animals without testosterone replacement indicates that the pro-aggressive effect of pCPA [25] is relevant only in the presence of testosterone. Our results are compatible with previous studies showing long-term administration of a lower dose of pCPA to gonadally intact rats to enhance rather than mask aggression induced by the exposure to high doses of anabolic androgens [13,35].

Where in the brain such a modulatory influence of serotonin on testosterone-dependent aggression takes place remains unknown. The medial amygdala, the hypothalamus, the prefrontal cortex and the lateral septum are however likely candidate areas, since these sites are all known to be involved in the regulation of aggression, and since they all display marked density of both sex steroid receptors and serotonergic nerve terminals [36–38].

While serotonin has often been attributed a general anti-aggressive influence, some authors have questioned this stance [8,39]. Instead, it has been suggested that a specific role for serotonin is to terminate aggression when the objectives of the aggressive behavior have been met [40], hence making the aggression functional and goal-directed. According to this theory, an impaired serotonergic transmission should primarily result in so-called maladaptive aggression, i.e. by an increase in the number of attacks rather than threats [41]. While we could not address this possibility in GDX mice not receiving testosterone, since pCPA failed to reinstate aggression in these animals, we did indeed observe a pattern of disinhibited attack behavior, i.e. a relative increase in the percentage of time spent on attacks without a corresponding increase in time spent on threats, in testosterone-treated GDX mice. Notably, an almost identical effect of pCPA was observed also in the gonadally intact wild-type mice of experiment II. Of note is that treatment with pCPA also led to shortened attack latency, which may also be interpreted as a sign of maladaptive aggression [42] in experiment II, with a similar trend, though not statistically significant, also in experiment I. It deserves to be mentioned that an increase in aggression was observed following the administration of the serotonin depleting agent in spite of the fact that both saline-treated GDX+testosterone mice and wild type animals displayed a relatively high amount of aggression as compared to what has been reported in some earlier studies [e.g. 25].

The pro-aggressive effect of testosterone is to a high extent mediated by ERs but may require ARs as well. Supporting an involvement of the latter, we could confirm the result of previous studies [28,29] showing AR^NesDel^ mice to display reduced aggression; since these animals display elevated testosterone and estrogen levels [28], and for this reason can be expected to display enhanced central ER activation, this observation clearly suggest that not only ER activation, but also AR activation, is required for normal male aggression to be at hand. Likewise, our observation, though based on a small number of animals, that pCPA failed to enhance aggression also in these mice, suggests that ER activation is not sufficient for serotonin depletion to provoke aggression, but that AR activation is required as well. Is should however be noted that brain ARs are inactivated also during development in these KO animals; if it is an early organizational effect of AR activation, or AR activation in the adult animal, that is necessary for pCPA to induce aggression, hence cannot be concluded from the present study.

A limitation of this study is that, for practical reasons, the number of AR^NesDel^ mice administered pCPA and saline, respectively, was low. However, given that PCPA did not elicit any aggression in any of the AR^NesDel^ mice tested, it is highly unlikely that expanding the group would have led to a markedly different outcome.

In conclusion, our data suggest that androgen-induced aggression is not masked by serotonin depletion, but, on the contrary, enhanced by pCPA, suggesting that testosterone does not exert its pro-aggressive effect by reducing serotonergic output. Instead the influence of serotonin on testosterone-induced aggression seems to be exerted by a parallel inhibitory pathway, the purpose of which seems to be to dampen maladaptive androgen-dependent aggression.

## References

[1] Kimura T, Hagiwara Y (1985) Regulation of urine marking in male and female mice: effects of sex steroids. Horm Behav 19: 64–70. 10.1016/0018-506X(85)90006-6 4038966

[2] Bing O, Heilig M, Kakoulidis P, Sundblad C, Wiklund L, Eriksson E (1998) High doses of testosterone increase anticonflict behaviour in rat. Eur Neuropsychopharmacol 8: 321–323. 10.1016/S0924-977X(97)00095-3 9928924

[3] Svensson A (2003) Testosterone treatment induces behavioral disinhibition in adult male rats. Pharmacol Biochem Behav 75: 481–490. 10.1016/S0091-3057(03)00137-0 12873641

[4] Wu M V, Shah NM (2011) Control of masculinization of the brain and behavior. Curr Opin Neurobiol 21: 116–123. 10.1016/j.conb.2010.09.014 20970320 PMC3046257

[5] Nelson RJ, Chiavegatto S (2001) Molecular basis of aggression. Trends Neurosci 24: 713–719. 10.1016/S0166-2236(00)01996-2 11718876

[6] Batty J, Meyerson BJ (1980) The effects of p-Chlorophenylalanine, fenfluramine and α-methyltyrosine on marking responses in the male Mongolian gerbil (Meriones unguiculatus). Pharmacol Biochem Behav 12: 181–184. 10.1016/0091-3057(80)90352-4 6246542

[7] Olivier B, Chan JSW, Snoeren EM, Olivier JDA, Veening JG, Vinkers CH, et al (2011) Differences in sexual behaviour in male and female rodents: role of serotonin. Curr Top Behav Neurosci 8: 15–36. 10.1007/7854_2010_116 21374021

[8] De Boer SF, Caramaschi D, Natarajan D, Koolhaas JM (2009) The vicious cycle towards violence: focus on the negative feedback mechanisms of brain serotonin neurotransmission. Front Behav Neurosci 3: 52 10.3389/neuro.08.052.2009 19949469 PMC2784299

[9] Sundblad C, Eriksson E (1997) Reduced extracellular levels of serotonin in the amygdala of androgenized female rats. Eur Neuropsychopharmacol 7: 253–259. 10.1016/S0924-977X(97)00031-X 9443656

[10] Zhang L, Ma W, Barker J., Rubinow D (1999) Sex differences in expression of serotonin receptors (subtypes 1A and 2A) in rat brain: a possible role of testosterone. Neuroscience 94: 251–259. 10.1016/S0306-4522(99)00234-1 10613515

[11] Ambar G, Chiavegatto S (2009) Anabolic-androgenic steroid treatment induces behavioral disinhibition and downregulation of serotonin receptor messenger RNA in the prefrontal cortex and amygdala of male mice. Genes Brain Behav 8: 161–173. 10.1111/j.1601-183X.2008.00458.x 19055689

[12] Martinez-Conde E, Leret ML, Diaz S (1985) The influence of testosterone in the brain of the male rat on levels of serotonin (5-HT) and hydroxyindole-acetic acid (5-HIAA). Comp Biochem Physiol C 80: 411–414. 10.1016/0742-8413(85)90077-5 2408817

[13] Keleta YB, Lumia AR, Anderson GM, McGinnis MY (2007) Behavioral effects of pubertal anabolic androgenic steroid exposure in male rats with low serotonin. Brain Res 1132: 129–138. 10.1016/j.brainres.2006.10.097 17194457

[14] Daly RC, Su T-P, Schmidt PJ, Pickar D, Murphy DL, Rubinow DR (2001) Cerebrospinal Fluid and Behavioral Changes After Methyltestosterone Administration. Arch Gen Psychiatry 58: 172 10.1001/archpsyc.58.2.172 11177119

[15] Näslund J, Studer E, Nilsson K, Westberg L, Eriksson E (2013) Serotonin depletion counteracts sex differences in anxiety-related behaviour in rat. Psychopharmacology (Berl) 230: 29–35. 10.1007/s00213-013-3133-6 23681161

[16] Liu Y, Jiang Y, Si Y, Kim J-Y, Chen Z, Rao Y (2011) Molecular regulation of sexual preference revealed by genetic studies of 5-HT in the brains of male mice. Nature 472: 95–99. 10.1038/nature09822 21441904 PMC4094133

[17] Bernhardt PC (1997) Influences of Serotonin and Testosterone in Aggression and Dominance: Convergence With Social Psychology. Curr Dir Psychol Sci 6: 44–48. 10.1111/1467-8721.ep11512620

[18] Kuepper Y, Alexander N, Osinsky R, Mueller E, Schmitz A, Netter P, et al (2010) Aggression—interactions of serotonin and testosterone in healthy men and women. Behav Brain Res 206: 93–100. 10.1016/j.bbr.2009.09.006 19747510

[19] Bethea CL, Reddy AP, Robertson N, Coleman K (2013) Effects of aromatase inhibition and androgen activity on serotonin and behavior in male macaques. Behav Neurosci 127: 400–414. 10.1037/a0032016 23506438 PMC3910396

[20] Birger M, Swartz M, Cohen D, Alesh Y, Grishpan C, Kotelr M (2003) Aggression: the testosterone-serotonin link. Isr Med Assoc J 5: 653–658. 14509157

[21] Higley JD, Mehlman PT, Poland RE, Taub DM, Vickers J, Suomi SJ, et al (1996) CSF testosterone and 5-HIAA correlate with different types of aggressive behaviors. Biol Psychiatry 40: 1067–1082. 10.1016/S0006-3223(95)00675-3 8931909

[22] Bonson KR, Johnson RG, Fiorella D, Rabin RA, Winter JC (1994) Serotonergic control of androgen-induced dominance. Pharmacol Biochem Behav 49: 313–322. 10.1016/0091-3057(94)90427-8 7529925

[23] Cologer–Clifford A, Simon NG, Richter ML, Smoluk SA, Lu S (1998) Androgens and Estrogens Modulate 5-HT1A and 5-HT1B Agonist Effects on Aggression. Physiol Behav 65: 823–828. 10.1016/S0031-9384(98)00240-6 10073487

[24] Koe BK, Weissman A (1966) p-Chlorophenylalanine: a specific depletor of brain serotonin. J Pharmacol Exp Ther 154: 499–516. 5297133

[25] Chiavegatto S, Dawson VL, Mamounas LA, Koliatsos VE, Dawson TM, Nelson RJ (2001) Brain serotonin dysfunction accounts for aggression in male mice lacking neuronal nitric oxide synthase. Proc Natl Acad Sci U S A 98: 1277–1281. 10.1073/pnas.031487198 11158630 PMC14745

[26] Ogawa S, Lubahn DB, Korach KS, Pfaff DW (1997) Behavioral effects of estrogen receptor gene disruption in male mice. Proc Natl Acad Sci U S A 94: 1476–1481. 9037078 10.1073/pnas.94.4.1476 PMC19816

[27] Sato T, Matsumoto T, Kawano H, Watanabe T, Uematsu Y, Sekine K, et al (2004) Brain masculinization requires androgen receptor function. Proc Natl Acad Sci U S A 101: 1673–1678. 10.1073/pnas.0305303101 14747651 PMC341816

[28] Raskin K, de Gendt K, Duittoz A, Liere P, Verhoeven G, Tronche F, et al (2009) Conditional inactivation of androgen receptor gene in the nervous system: effects on male behavioral and neuroendocrine responses. J Neurosci 29: 4461–4470. 10.1523/JNEUROSCI.0296-09.2009 19357272 PMC6665718

[29] Juntti SA, Tollkuhn J, Wu MV, Fraser EJ, Soderborg T, Tan S, et al (2010) The androgen receptor governs the execution, but not programming, of male sexual and territorial behaviors. Neuron 66: 260–272. 10.1016/j.neuron.2010.03.024 20435002 PMC2923659

[30] Barkley MS, Goldman BD (1977) The effects of castration and Silastic implants of testosterone on intermale aggression in the mouse. Horm Behav 9: 32–48. 10.1016/0018-506X(77)90048-4 561020

[31] De Gendt K, Swinnen JV, Saunders PTK, Schoonjans L, Dewerchin M, Devos A, et al (2004) A Sertoli cell-selective knockout of the androgen receptor causes spermatogenic arrest in meiosis. Proc Natl Acad Sci U S A 101: 1327–1332. 10.1073/pnas.0308114100 14745012 PMC337052

[32] Bourghardt J, Wilhelmson ASK, Alexanderson C, De Gendt K, Verhoeven G, Krettek A, et al (2010) Androgen receptor-dependent and independent atheroprotection by testosterone in male mice. Endocrinology 151: 5428–5437. 10.1210/en.2010-0663 20861231

[33] Natarajan D, de Vries H, Saaltink D-J, de Boer SF, Koolhaas JM (2009) Delineation of violence from functional aggression in mice: an ethological approach. Behav Genet 39: 73–90. 10.1007/s10519-008-9230-3.34 18972199 PMC9823070

[34] Gao X, Dluzen DE (2001) The effect of testosterone upon methamphetamine neurotoxicity of the nigrostraiatal dopaminergic system. Brain Res 892: 63–69. 11172749 10.1016/s0006-8993(00)03221-2

[35] Kubala KH, McGinnis MY, Anderson GM, Lumia AR (2008) The effects of an anabolic androgenic steroid and low serotonin on social and non-social behaviors in male rats. Brain Res 1232: 21–29. 10.1016/j.brainres.2008.07.065 18692488

[36] Lu SF, McKenna SE, Cologer-Clifford A, Nau EA, Simon NG (1998) Androgen receptor in mouse brain: sex differences and similarities in autoregulation. Endocrinology 139: 1594–1601. 10.1210/endo.139.4.5863 9528939

[37] Cologer-Clifford A, Simon NG, Lu SF, Smoluk SA (1997) Serotonin agonist-induced decreases in intermale aggression are dependent on brain region and receptor subtype. Pharmacol Biochem Behav 58: 425–430. 10.1016/S0091-3057(97)00295-5 9300602

[38] Michelsen KA, Schmitz C, Steinbusch HWM (2007) The dorsal raphe nucleus—from silver stainings to a role in depression. Brain Res Rev 55: 329–342. 10.1016/j.brainresrev.2007.01.002 17316819

[39] Olivier B, van Oorschot R (2005) 5-HT1B receptors and aggression: a review. Eur J Pharmacol 526: 207–217. 10.1016/j.ejphar.2005.09.066 16310769

[40] Takahashi A, Quadros IM, de Almeida RMM, Miczek KA (2011) Brain serotonin receptors and transporters: initiation vs. termination of escalated aggression. Psychopharmacology (Berl) 213: 183–212. 10.1007/s00213-010-2000-y 20938650 PMC3684010

[41] Caramaschi D, de Boer SF, de Vries H, Koolhaas JM (2008) Development of violence in mice through repeated victory along with changes in prefrontal cortex neurochemistry. Behav Brain Res 189: 263–272. 10.1016/j.bbr.2008.01.003 18281105

[42] Natarajan D, Caramaschi D (2010) Animal violence demystified. Front Behav Neurosci 4: 9 10.3389/fnbeh.2010.00009 20407576 PMC2854525

